# Neuroscience of human social interactions and adult attachment style

**DOI:** 10.3389/fnhum.2012.00212

**Published:** 2012-07-17

**Authors:** Pascal Vrtička, Patrik Vuilleumier

**Affiliations:** ^1^Swiss Center for Affective Sciences, University of GenevaGeneva, Switzerland; ^2^Laboratory for the study of Emotion Elicitation and Expression (E3 Lab), Department of Psychology, FPSE, University of GenevaGeneva, Switzerland; ^3^Laboratory for Neurology and Imaging of Cognition, Department of Neuroscience, University Medical Center, University of GenevaGeneva, Switzerland

**Keywords:** adult attachment style, functional neuroanatomical framework, human social interactions, cognitive affective neuroscience, emotional versus cognitive mentalization

## Abstract

Since its first description four decades ago, attachment theory (AT) has become one of the principal developmental psychological frameworks for describing the role of individual differences in the establishment and maintenance of social bonds between people. Yet, still little is known about the neurobiological underpinnings of attachment orientations and their well-established impact on a range of social and affective behaviors. In the present review, we summarize data from recent studies using cognitive and imaging approaches to characterize attachment styles and their effect on emotion and social cognition. We propose a functional neuroanatomical framework to integrate the key brain mechanisms involved in the perception and regulation of social emotional information, and their modulation by individual differences in terms of secure *versus* insecure (more specifically avoidant, anxious, or resolved *versus* unresolved) attachment traits. This framework describes how each individual's attachment style (built through interactions between personal relationship history and predispositions) may influence the encoding of approach *versus* aversion tendencies (safety *versus* threat) in social encounters, implicating the activation of a network of subcortical (amygdala, hippocampus, striatum) and cortical (insula, cingulate) limbic areas. These basic and automatic affective evaluation mechanisms are in turn modulated by more elaborate and voluntary cognitive control processes, subserving mental state attribution and emotion regulation capacities, implicating a distinct network in medial prefrontal cortex (mPFC), superior temporal sulcus (STS), and temporo-parietal junction (TPJ), among others. Recent neuroimaging data suggest that affective evaluation is decreased in avoidantly but increased in anxiously attached individuals. In turn, although data on cognitive control is still scarce, it points toward a possible enhancement of mental state representations associated with attachment insecurity and particularly anxiety. Emotion regulation strategies such as reappraisal or suppression of social emotions are also differentially modulated by attachment style. This research does not only help better understand the neural underpinnings of human social behavior, but also provides important insights on psychopathological conditions where attachment dysregulation is likely to play an important (causal) role.

## Introduction

In mammals, including humans, attachment is a major dimension of behavior that can come into play in several domains (Fisher et al., 2006). This includes bond formation and maintenance between children and parents (parental care), love and sexual fidelity between long-term partners (partner attachment), but also various social links between individuals in a group. How much people value and react to interactions with others is undoubtedly a major ingredient of human life and emotions. In recent years, important progresses have been achieved by neuroscience research concerning the brain circuits involved in basic sexual and parental bonding (Insel and Young, 2001), as well as the close functional interactions between social and emotional/motivational systems in the brain (Lieberman, 2007), but the neural processes subserving affective attachment of humans to others in various conditions still remain to be elucidated.

The notion of attachment is a central feature of a prominent theoretical framework of social-emotional behavior in developmental psychology, known as attachment theory (AT) (Bowlby, 1969, 1982). This framework relies on the assumption that every human being is born with an innate attachment system, whose biological function is to obtain or maintain proximity to significant others in times of need or presence of threats, and thus to regulate support seeking behavior. Such a function is crucial for survival in early life, as a child cannot live on its own without the care of his/her primary attachment figure—mainly the mother. This is especially vital in mammals, as the mother is the main resource for food, and even more so in humans, because the time span during which an offspring is dependent on external care is particularly long. Importantly, however, AT suggests that repeated interactions with attachment figures (e.g., parents), and the responses of the latter to the proximity seeking attempts of the child, will induce the formation of differential cognitive schemes for representing the self and others, and for behaving in interpersonal relationships later on in life. These processes are thought to lead to the establishment of so-called internal working models of attachment (IWMs), encoding expectations of care and allowing a “*mental simulation and prediction of likely outcomes of various attachment behaviors*” (Mikulincer and Shaver, 2007) when interacting with significant social partners. This will then constitute the foundation of a person's individual attachment style, which remains fairly stable into adulthood and may provide a template for determining how people perceive and react during various types of social encounters. Thus, although adult attachment style (AAS) may influence response patterns during close relationships with other individuals (e.g., romantic partners), it is considered to also operate during interactions or social appraisals with unknown people, as well as during a range of different emotional situations throughout life (Niedenthal et al., 2002; Fraley et al., 2006; Mikulincer and Shaver, 2007). The impact of individual differences in AAS on social and affective functioning is therefore thought to go far beyond the specific behaviors associated with parental and partner attachment (Fisher et al., 2006).

Although very prominent in developmental psychology (Mikulincer and Shaver, 2007) and some psychopathological theories (Fonagy and Luyten, 2009), the social-affective phenomena associated with attachment style as well as their impact on human behaviors and their neural mechanisms have only rarely been investigated in a human neuroscience perspective. The current review therefore aims at providing an overview of recent investigations that combined an AT perspective with cognitive and neurobiological approaches. Doing so may offer novel and promising avenues for future research, not only to better understand normal social behaviors in humans, including individual differences in AAS; but also to illuminate some conditions or pathologies associated with disturbances in social emotional functioning, such as autism (Andari et al., 2010), schizophrenia (Abdi and Sharma, 2004; Marwick and Hall, 2008), borderline personality (Fonagy and Luyten, 2009; Fonagy et al., 2011), or violence and sociopathy (Decety et al., 2009; Blair et al., 2011a,b). In this review, we will first introduce the general theoretical aspects of AT and discuss how it may offer a fruitful framework in social cognitive and affective neuroscience. We will then mainly focus on the functional neurobiological mechanisms of social and affective processing that may underlie individual differences in attachment style.

## Attachment theory

Distinct individual profiles in attachment style have been described and can be identified in adults by specific questionnaires or semi-structured interviews (see Mikulincer and Shaver, 2007 for an overview). In the case of an available and responding attachment figure providing a “secure base” for restoring emotional balance in times of distress, a positive model of others linked with supportiveness and trustworthiness can be developed, paired with positive self-attributes such as worthy, competent, and lovable. This allows the formation of a *secure attachment style*. In contrast, an insecure attachment style will emerge if attachment figures are repeatedly experienced as unresponsive or inconsistent in their responses in times of need and stress. Two major patterns of insecurity have been classically distinguished: either avoidant or anxious attachment, associated with the establishment of attachment system *de-activation* or *hyper-activation* as secondary attachment strategies, respectively, (Mikulincer and Shaver, 2007).

In the case of *attachment avoidance*, proximity seeking is perceived as futile or even dangerous because of the distress felt by failing to achieve proximity to an attachment figure. Consequently, avoidant individuals develop a dismissive approach to and a negative model of others, operating through the denial of positive traits in others. They disavow needs for attachment, avoid affective closeness and intimacy, but seek independence with the goal to prevent the felt rejection by others. Concomitantly, they tend to suppress negative aspects of the self and boost their positive features instead, leading to the emergence of a positive self-model. In addition, attachment avoidance is associated with a preferential use of (expressive) suppression to regulate emotions (Mikulincer and Shaver, 2007), allowing the individual to keep the attachment system in a low activation state and to prevent others of perceiving their internal emotional states (Vrticka et al., 2012a).

The other main form of insecurity is *attachment anxiety*, where a perceived failure to handle threats autonomously will encourage subjects to intensify their support-seeking attempts despite the fact that attachment figures are experienced as inconsistent. In this case, others are still viewed as (partly) positive due to the desire for attention and protection. However, repeated experience of rejection leads to an increased sense of helplessness and vulnerability, paired with doubts about self-worth and -efficacy, leading to a negative internal model of the self and poor self-esteem. Such individuals become highly vigilant to potential threats and rejections. This style is also thought to imply a distinctive emotion regulation strategy, with preferential use of re-appraisal but in the “wrong” direction: instead of decreasing the impact of negative emotions, these subjects actually tend to intensify the impact of negative social signals due to their hypersensitivity to the latter (Bartholomew, 1990; Bartholomew and Horowitz, 1991; Griffin and Bartholomew, 1994; Mikulincer and Shaver, 2007).

Besides these three main categories of secure, avoidant, and anxious attachment styles, a fourth attachment orientation has been proposed, referred to as *fearful* or *disorganized* (Main et al., 1985; Bartholomew and Horowitz, 1991; Griffin and Bartholomew, 1994; Mikulincer and Shaver, 2007). It is either characterized by the presence of both avoidant and anxious attachment traits, reflecting negative models for both the self and others, or by disoriented attachment behaviors indicating the lack of a coherent attachment strategy. The latter type is also called *unresolved* attachment, in contrast to the resolved/organized attachment orientations corresponding to the secure, avoidant, or anxious styles. Such a dissociation between resolved *versus* unresolved attachment categories is particularly prominent in psychopathology research, where it has been proposed that attachment dysregulations in terms of an unresolved attachment orientation might lay at the core of some emotional disturbances, including borderline personality disorder (BPD; Fonagy and Luyten, 2009), as well as schizoaffective disorder, bipolar disorder, and major depression (Berry et al., 2007).

On the ground of such descriptions of secure and insecure AAS, Mikulincer and Shaver (2007) have proposed that an extensive list of human social behaviors might be importantly modulated by these psychological traits. This includes (1) romantic and sexual behavior, (2) self-regulation and personal growth, (3) emotion regulation and coping, (4) interpersonal regulation, as well as (5) family functioning and parental care. In addition, AAS may also influence more general behaviors related to affect and motivation, including pain and medical care (Meredith et al., 2006; Hooper et al., 2012). Thus, attachment dysregulations are nowadays recognized as important contributors to various emotional and social disturbances, a fact which bolsters the need of better understanding their cognitive underpinnings as well as their neural substrates.

However, the current distinction of AAS into three, four, or even five main categories has been questioned by some researchers who proposed instead to conceive these individual differences along a single continuum of emotional security (e.g., Fraley and Spieker, 2003a). For example, attachment and affective social behaviors might be mapped on two independent dimensions of anxiety and avoidance (Bartholomew and Horowitz, 1991), with the secure style corresponding to both low anxiety and low avoidance, and the disorganized style to high traits in both anxiety and avoidance. Thus, it remains to be clarified whether individual differences in AAS mainly refer to a true taxonomy of personality traits or to some underlying mechanisms that might result in distinct patterns of attachment behaviors. Nevertheless, this issue does not undermine the general assumptions of AT (Waters and Beauchaine, 2003), and both classification schemes seem equally useful for analyzing individual differences in attachment security and social interactions (Fraley and Spieker, 2003b).

Furthermore, some aspects of AAS might partly overlap with other important psychological dimensions associated with individual personality traits, such as neuroticism, reward dependence, and novelty seeking (Chotai et al., 2005). Hence, it also remains to be better determined what the specificity of these different constructs really is. Importantly, functional neuroimaging studies might help to address this issue, for example by showing that differences in attachment anxiety and avoidance correlate with functional modulations in distinct brain systems. Moreover, some of these effects on brain activity may be specific to attachment traits and do not correlate with other personality or anxiety measures (see Vrtička et al., 2008; Vrticka et al., 2012a). Yet, as we describe below, we are only just beginning to unveil the cerebral architecture of various components that are potentially at play in the emotional and behavioral features of AAS.

## Attachment-related effects on behavior and cognition

The influence of individual differences in attachment style on emotion processing and social cognition has been extensively demonstrated in a wide range of behavioral experiments. The latter have generally examined how attachment style, alone or combined with tasks activating cognitive representations of attachment, may influence performance in vigilance, attentional monitoring, perceptual judgment, or memory for verbal material or emotional facial expressions. These effects illustrate the varieties of mental functions that are potentially modulated as a function of individual differences in AAS. Although the corresponding neural substrates are generally unknown, these behavioral effects provide an important cornerstone to identify processing stages influenced by attachment style, and to guide neurobiological investigations with brain imaging or other means. Below we briefly summarize behavioral findings related to different cognitive and affective domains, in order to provide a comprehensive overview of the field, but in subsequent sections concerning brain systems our review will more specifically focus on emotional and social domains.

### Attachment effects on emotion processing

Since attachment style is thought to influence individual responses to social affective cues, emotion processing has been explored in various task conditions, for different kinds of stimuli. A few studies examined the processing of emotional facial expressions in a movie morph paradigm (Niedenthal et al., 2002; Fraley et al., 2006), in which faces could change from neutral to happy, sad, or angry, and vice versa. The results showed that the detection of both the onset and offset of all emotional expressions was reported earlier by people with insecure attachment (anxiety, avoidance, or more general attachment insecurity). Remarkably, faces were from unknown people in these experiments, indicating that AAS can have profound influences on emotional appraisals even for unfamiliar social material during simple perceptual tasks.

Another investigation of emotion perception looked at more controlled processes by asking participants to make explicit ratings of pleasantness and arousal for video-clips with attachment-related content (Rognoni et al., 2008). The results showed that anxiously attached individuals rated negative emotions of fear and sadness as more arousing, as compared with secure individuals, whereas avoidantly attached participants rated positive emotions as less arousing. These findings are consistent with theoretical proposals postulating an enhanced responsiveness to negative social cues associated with anxious attachment, and a dismissal of positive interactions associated with avoidance. Similarly, in a recent behavioral experiment carried out in our own laboratory (Vrticka et al., 2012b), participants were asked to explicitly rate visual images depicting either positive or negative, and either social or nonsocial, scenes along scales of pleasantness, arousal, and control. Again, attachment avoidance was associated with a selective decrease of pleasantness ratings but only for positive social scenarios, whereas attachment anxiety was associated with both increased arousal and decreased control ratings for negative social scenarios specifically. These data underscore the selective impact of AAS on affective responses to social cues, rather than on more general emotional information.

### Attachment effects on selective attention

In addition to emotion, several studies examined how information processing may be biased by personal representations of attachment. For example, some studies showed that anxiously attached participants are faster to make lexical decisions in response to the names of their attachment figures (Mikulincer et al., 2002), and display a selective hypervigilance toward attachment names (Dewitte et al., 2007a,b). Likewise, attachment style can affect attention in a Stroop task when it is performed following exposure to threat- versus neutral-word primes (i.e., Mikulincer et al., 2002) or attachment-related versus -unrelated words (i.e., Edelstein, 2006). In one of these studies (Mikulincer et al., 2002), the Stroop task was made of names of attachment figures and measured their degree of accessibility (by naming latencies) in threatening and nonthreatening contexts. Response times were slower in anxiously attached participants in both the neutral and threat prime conditions, but faster in avoidantly attached participants specifically after presentation of the threat-word primes. These data were taken to suggest that attachment anxiety leads to a heightened processing of attachment-related information in general, whereas attachment avoidance entails opposite effects (i.e., suppression of processing) during negative contexts specifically. Another study (Edelstein, 2006) confirmed these findings for avoidant attachment style by showing that emotional Stroop interference is reduced for attachment-related words. This study also revealed that such inhibition of attention to potentially threatening information requires cognitive effort because it was attenuated under conditions with simultaneous increase in cognitive load.

Other experiments tested for attention effects by using a dot-probe task in which participants were presented with either pairs of positive or negative attachment-related or attachment-unrelated words (Dewitte et al., 2007b), or pairs of different kinds of known or unknown names (Dewitte et al., 2007a). The results revealed that both avoidantly and anxiously attached individuals were characterized by preferential orienting of attention away from negative attachment-related words, relative to secure individuals. In addition, anxious attachment was also associated with an attentional bias toward positive and negative attachment-related (versus attachment-unrelated) names (Mikulincer and Shaver, 2007). Taken together, such findings suggest that negative attachment-related information might be feared in case of highly anxious and/or highly avoidant attachment traits, but only attachment anxiety to lead to an enhanced representation of attachment signals under threatening circumstances.

### Attachment effects on memory

A third line of studies examining the impact of attachment style on cognition has focused on memory processes, using forced-choice recall of emotionally-laden drawings (Kirsh, 1996), free recall for positive, neutral, or threatening words (Van Emmichoven et al., 2003), as well as an operation-word span task including neutral, emotional, and attachment related words during working memory performance (Edelstein, 2006). The first of these studies reported that avoidantly attached individuals remembered depictions of anger better than securely or anxiously attached participants, whereas the second found better recall for threatening words in securely attached compared to insecurely attached participants. In the working memory domain, deficits were observed in avoidantly attached participants for both positive and negative attachment-related stimuli (Edelstein, 2006). The latter findings for working memory performance are highly consistent with the proposal that avoidant individuals tend to defensively inhibit the processing of potentially distressing information (Edelstein, 2006). However, in contrast, data from the memory recall tasks are partly divergent and not directly predicted by AT (Mikulincer and Shaver, 2007). More research is therefore needed to clarify the effect of individual attachment traits on various stages of memory functioning.

Taken together, the existing behavioral findings clearly show that individual differences in AAS correlate with difference in a range of cognitive and affective processes, particularly in attachment-relevant or social contexts. Moreover, these effects may act on different kinds of functions, operating both at a rather automatic or implicit (even unconscious) level and at a more voluntary or explicit (conscious) levels of processing. However, the exact neural mechanisms involved in these effects remain largely unexplored, although general models of social cognition and emotion processing (Lieberman, 2007) suggest that they should implicate several distinct brain circuits.

## Neuroscience of human social interactions from an attachment theory perspective: the role of automatic affective appraisals

**Figure 1:**
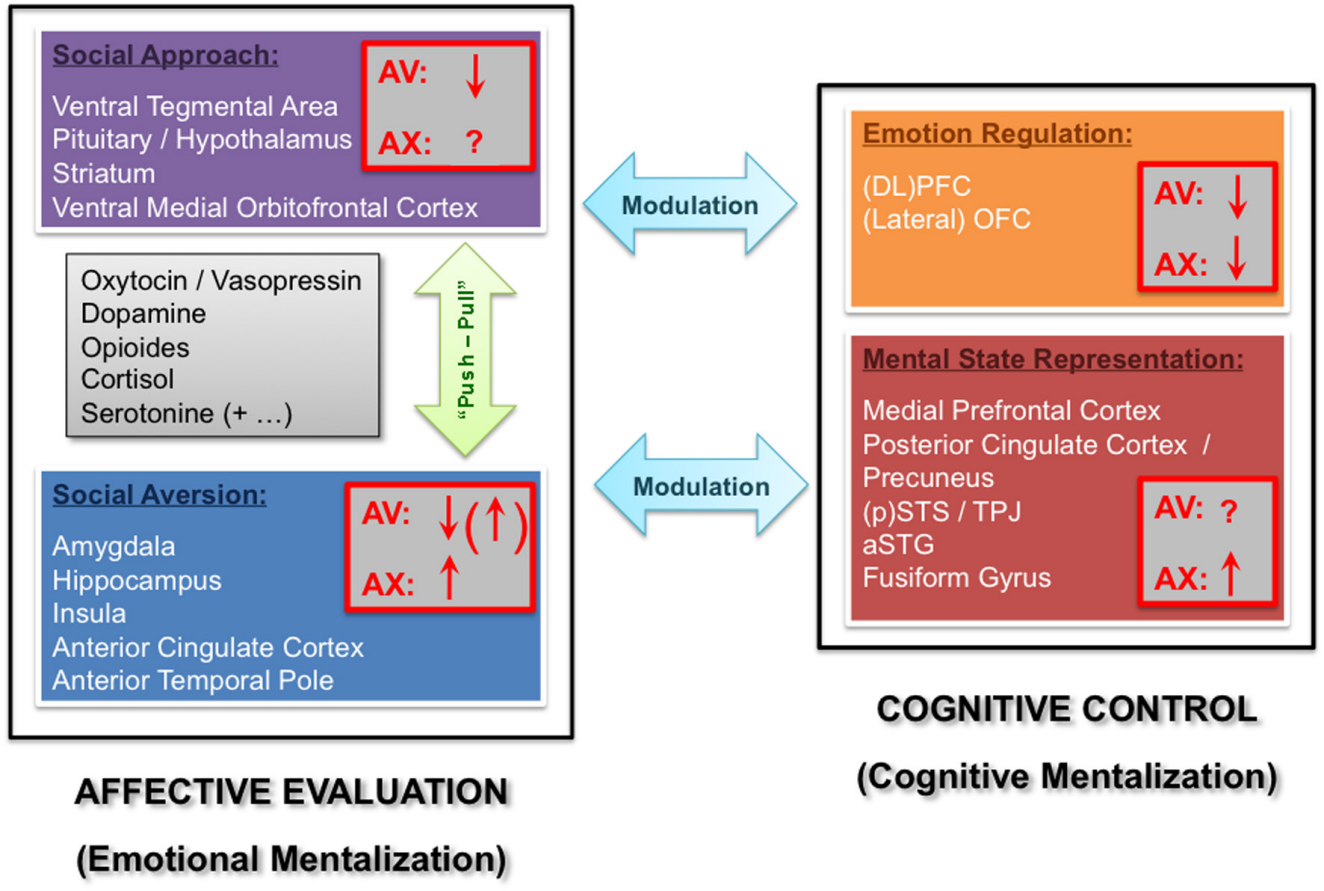
**Functional neuroanatomical model of the influence of adult attachment style on social processing.** Two core component networks mediate relatively automatic affective evaluations versus more controlled cognitive processes, broadly corresponding to emotional versus cognitive mentalization mechanisms proposed in other models (Fonagy and Luyten, 2009). The affective evaluation component further comprises social approach (purple) versus aversion (blue) systems, whereas the cognitive control component comprises distinct systems implicated in emotion regulation (orange) and mental state representation (red). We assume “push-pull” effects between approach versus aversion modules (green arrow), which might be jointly influenced by learning as well as genetic factors (e.g., neuromodulator systems listed in the gray box). In addition, more complex reciprocal influence may exist between the affective evaluation and cognitive control components (turquoise arrows). The possible influence of attachment avoidance (AV) or anxiety (AX) on activity of each of these networks is depicted by (downward or upward) arrows (red boxes) representing relative hypo- or hyper-activation, respectively. For details, please refer to text. (DL)PFC = (dorsolateral) prefrontal cortex; OFC = orbitofrontal cortex; (p)STS = (posterior) superior temporal sulcus; TPJ = temporo-parietal junction; aSTG = anterior superior temporal gyrus.

As mentioned above, the neuroscientific investigation of attachment in humans has just recently begun. To date, only a handful of studies have probed brain systems activated during social interactions or emotional tasks from an AT perspective, and even fewer explored the influence of individual differences in secure or insecure AASs. The following sections will provide an overview of recent findings from these studies, specifically those focusing on the neural substrates of human attachment as well as those exploring other relevant social functions with a neuroscience approach. While doing so, we will organize the putative mechanisms modulated by AAS in a general framework (see Figure 1), with distinct functional components based on both (1) current cognitive and affective neuroscience models, and (2) modern views on AT. Specifically, we will distinguish brain systems modulated by individual attachment orientations that belong, on the one hand, to networks associated with basic affective evaluation processes, such as threat or reward, and on the other hand, networks that are associated instead with cognitive control and mentalizing abilities, such as a theory of mind, self-reflection, and emotion regulation.

### Social approach

Several models of emotion and social cognition (e.g., Phillips et al., 2003a,b; Lieberman, 2007; LeDoux, 2012) include core processes subserving rapid or automatic (sometimes even unconscious) processing of information in terms of safety *versus* danger, which are intrinsically linked with behavioral tendencies to either approach or avoid a stimulus. Automatic appraisals of danger and safety may thus also apply to socially relevant cues, and guide adaptive behaviors in a quasi “reflexive” manner. This concept draws upon the *phylogenetic perspective of social engagement and attachment* proposed by Porges (Porges, 2003). This author suggested that in order to achieve prosocial ends, evolution had to counterbalance the asocial tendencies of more primitive survival-enhancing systems, especially sympathetic fight-or-flight circuits. In other words, there might be a dynamic balance, or a “*push-pull*” mechanism, between activity in a threat-sensitive system motivating social aversion, on the one hand, and an attachment system that promotes a sense of safety through close social interactions, on the other hand (for a summary, see MacDonald and MacDonald, 2011). Both the social approach and aversion components might potentially be modulated by differences in attachment style (see Figure 1).

In this perspective, a fundamental hypothesis about the social approach component is that it might build upon specific brain mechanisms related to the “*neuroception of safety*” (Porges, 2003), which assumes that (mutual) social interactions are innately rewarding and thus counteracting fear tendencies. Such a view converges with research associating activations in dopaminergic brain areas [including the ventral tegmental area, substantia nigra, striatum, and medial orbitofrontal cortex (OFC)] with many different kinds of positive social emotions, as well as with the modulations of fear-circuits in the amygdala by dopaminergic inputs (Haber and Knutson, 2010). There is mounting evidence for a role of reward circuits and reinforcing processes in social approach and bonding from several recent functional and structural brain imaging investigations on maternal and romantic love (Lorberbaum et al., 1999; Nitschke et al., 2004; Ranote et al., 2004; Aron et al., 2005; Fisher et al., 2005, 2006; Gobbini and Haxby, 2007; Sander et al., 2007; Swain et al., 2007; Zeki, 2007; Noriuchi et al., 2008; Strathearn et al., 2008; Lenzi et al., 2009; Minagawa-Kawai et al., 2009; Kim et al., 2010a,b, 2011; Xu et al., 2011), the perception of the mother's face in children (Minagawa-Kawai et al., 2009) as well as the experience of social reward (e.g., a smiling face paired with positive feedback after correct task performance) over and above positive feedback alone (Vrtička et al., 2008). Altogether, these data suggest that, under normal circumstances, social interactions with beloved ones (children, parents, partners), friends, or any “significant” (e.g., contextually relevant) other person with a cooperative relationship (e.g., joint task), are all associated with the experience of positive emotions and increased activity in the reward circuits. This could contribute to promoting expectations of positive social outcomes, and thus in turn enhance approach-related attachment behaviors and a feeling of safety.

The recruitment of this positive reinforcement mechanism has been found to be strongly influenced by individual differences in AAS. On the one hand, individuals with a *secure attachment style* (or other measures indirectly suggesting a secure attachment orientation) were observed to show stronger activation, or display increased gray matter volume, in the reward network as well as other interconnected regions such as the hypothalamus or OFC. For instance, the ventral striatum differentially activates in secure mothers seeing images of their own babies with a smiling or a crying facial expression (Strathearn et al., 2009). Also, in mothers who score higher on the mother positive perception subscale of the Yale inventory of parental thoughts and actions, there is not only increased gray matter volume, but also more activity in OFC in response to infant cries (Kim et al., 2010a). Likewise, activity in ventral striatum and ventral tegmental area is greater in secure than insecure individuals when receiving positive social feedback (praise by others) after a correct performance in a perceptual game (Vrtička et al., 2008). Thus, in securely attached individuals, (mutual) social interactions indeed seem generally to be associated with more positive emotion experiences and stronger signals of reward.

**Figure 2:**
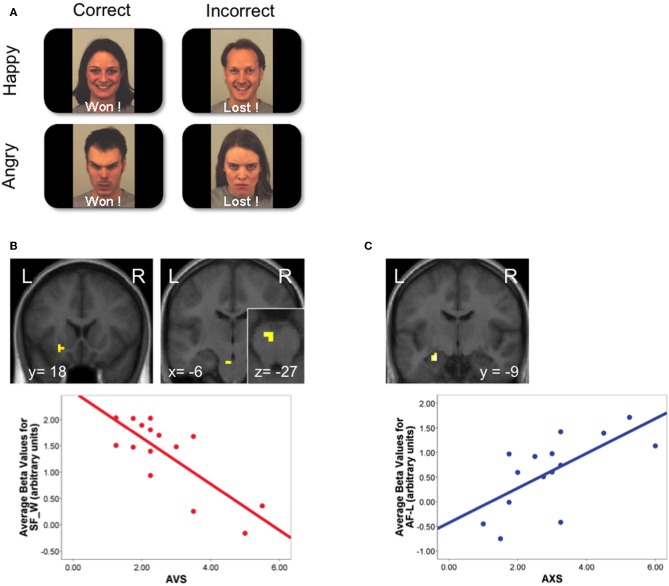
**Modulation of social aversion and approach activations by adult attachment style.** Adapted from Vrtička et al. (2008). **(A)** Participants performed a visual task, while receiving feedback from virtual partners about their performance. Feedback was composed of words reflecting outcome (“Won” if correct and “Lost” if incorrect response was given), associated with either smiling or angry faces, inducing the perception of supportive “friends” (congruent word-face combinations) or hostile “opponents” (incongruent combinations). **(B)** *Top:* reward-related areas (left: ventral striatum; right: ventral tegmental area) were activated during the perception of positive social feedback (“Won” paired with a smiling face; SF-W), but this effect was modulated by avoidant attachment style. *Bottom:* negative relation between avoidant attachment style (AVS) scores and the ventral striatum response. **(C)** *Top:* the central amygdala was activated by the perception of social punishment (“Lost” paired with an angry face; AF-L), and this effect was modulated by anxious attachment style. *Bottom*: positive relation between anxious attachment style (AXS) scores and amygdala response. BOLD signal is depicted in arbitrary units.

On the other hand, these positive responses are much weaker or even absent in individuals with an *avoidant attachment style*. This was first demonstrated in a recent study (Vrtička et al., 2008) where different faces were presented with different expressions (smiling or angry) to convey either positive/supportive or negative/hostile feedback about current task performance in a pseudo-interactive game context (see Figure 2A). Differential responses in ventral striatum and ventral tegmental area to the social nature of feedback (smiling face *versus* angry faces on winning trials) was inversely correlated with increasing scores on the avoidant attachment dimension (Vrtička et al., 2008; see Figure 2B). This pattern is further supported by the findings of very low ventral striatum and medial OFC activation in avoidantly *versus* securely attached mothers when seeing images of their own babies (Strathearn et al., 2009). An avoidant attachment style, which is thought to emerge due to early and/or repeated social interactions with an unresponsive attachment figure, and characterized by a negative model of others (Mikulincer and Shaver, 2007), therefore seems to entail a profound change in the social approach system (see Figure 1), leading to a reduction or lack of reward-related activity during positive social situations. It still remains to be determined whether such blunted responses in reward-related areas associated with avoidant attachment are primarily due to past experiences and learning mechanisms (so to speak as a result of down-regulation or desensitization), or whether they also have a partly genetic cause (i.e., receptor-gene polymorphisms, reduction of certain neurotransmitters, etc.), or whether they emerge as a combination of these two factors through gene-environment interactions (see below).

These neural findings dovetail nicely with behavioral evidence that avoidant individuals rate positive social information as less arousing and less pleasant, relative to securely attached individuals (Rognoni et al., 2008; Vrticka et al., 2012a). More generally, they also agree with some key assumptions put forward by AT, according to which avoidance is associated with the use of deactivating strategies to keep the attachment system in a very low recruitment state, although behavioral research has most often considered these effects in relation to the processing of negative rather than positive (social) content (Mikulincer and Shaver, 2007). The new findings therefore add to previous work by indicating that avoidantly attached people could appraise positive social interactions with less—or even no—intrinsically rewarding values, and perhaps fail to learn from positive social reinforcers. These notions may not only help refine AT but also provide new clues for therapeutic clinical strategies tailored to treat attachment disturbances.

By contrast, there is no evidence that *anxious attachment style* is associated with a modulation of neural processes related to social approach (Vrtička et al., 2008). As described in the next section, this attachment dimension seems primarily related to the appraisals of negative social cues, consistent with the assumptions of AT (Mikulincer and Shaver, 2007).

### Social aversion

According to the same phylogenetic perspective on social engagement and attachment as described above (Porges, 2003), the approach system should be in a dynamic balance (“*push-pull*”) with a distinct social aversion network (see Figure 1). In humans, appraisals of potential threats to the self may not only concern information that poses an immediate danger for survival or bodily integrity—such as physical pain or disgust—but brain systems responding to such general threats are also recruited when processing dangers of a more social kind. Thus, a set of regions typically associated with negative affect and fear responses are activated by various aversive social events including—amongst others—the perception of untrustworthiness of faces in the amygdala (Engell et al., 2007), stressful social situations in the hippocampus—as part of the HPA axis—(Foley and Kirschbaum, 2010), psychological pain and social rejection in insula and mid cingulate cortex (Eisenberger and Lieberman, 2004; Lamm et al., 2011), social emotional conflict in ventral anterior cingulate cortex (ACC) (Somerville et al., 2006; Koban et al., 2010), as well as the experience of sadness or grief in anterior temporal pole (ATP; Levesque et al., 2003; Kersting et al., 2009). Thus, appraisals of negative social contexts will trigger activity in a network of areas promoting aversion, withdrawal, or even defense responses.

Recent neuroimaging data suggests that both the functioning and structure of brain areas contributing to this social aversion component is modulated by a *secure attachment style* (see Figure 1). In a pioneer study in this field, securely attached married female participants (as assessed by the satisfaction subscale of the dyadic adjustment scale—measuring marital quality) were found to show less insula activation during both the anticipation and experience of electrical shocks while holding their partners hand, implying weaker distress reactions and more successful emotional support (Coan et al., 2006). Another study using structural MRI measures reported increases in gray matter volume in the amygdala in mothers at four months compared to one month postpartum, and this postpartum plasticity was correlated with scores on the maternal positive perception subscale of the Yale inventory of parental thoughts and actions (Kim et al., 2010a). Such findings suggest a progressive development of affective vigilance mechanisms in mothers who recently gave birth, a notion corroborated by previous findings showing highest amygdala activation in mothers for own *versus* other familiar or unknown children (Leibenluft et al., 2004). Therefore, a secure attachment orientation seems to be associated changes in key structures of the aversion system, presumably reflecting more differentiated and thus adaptive responses to social stimuli.

In individuals with *avoidant attachment*, partly similar effects have been observed, namely, a relative deactivation of brain areas associated with social aversion (see Figure 1). In a recent fMRI study using the cyberball paradigm (a virtual ball tossing game during which the participant see two people playing with a ball either including or excluding him/her into the game), which has previously been found to induce social rejection and “social pain” (Eisenberger et al., 2003), avoidantly attached individuals showed decreased activations in the anterior insula and dorsal ACC during social exclusion (Dewall et al., 2011). This was interpreted as reflecting the weaker social need for closeness and weaker distress elicited by social rejection in these individuals. Along the same lines, masked sad faces were found to induce weaker responses in the somatosensory cortex (BA 3) of avoidantly attached participants, compared to secure participants, which was attributed to their “habitual unwillingness to deal with partners' distress and needs for proximity” (Suslow et al., 2009). Avoidant attachment has also been related to weaker attentional and semantic processing of fearful faces in an EEG study (Zhang et al., 2008).

However, contrary to securely attached persons, such blunted responses to social negative contexts in avoidantly attached individuals are thought to result from deactivating strategies that suppress the recruitment of attachment processes in order to circumvent too strong emotional involvement (Mikulincer and Shaver, 2007), rather than from a more effective regulation of emotions during negative or stressful social interactions. This highlights the fact that emotional decreases in a given brain area might result from different causes, which might be difficult to interpret when considered alone. Moreover, an apparent decreased sensitivity to social rejection in avoidantly attached individuals may work well under normal circumstances, but tends to fail if the social emotional stimuli are too disturbing or intense (i.e., in insecure mothers seeing their baby with a crying facial expression—see Strathearn et al., 2009), or if the emotion suppression strategy usually referred to cannot be employed successfully—see Vrticka et al., 2012a and below). In fact, a different pattern of results, with higher rather than lower amygdala responses to emotional social stimuli, may be observed in avoidantly attached individuals in some conditions when emotion regulation strategies are constrained by specific task demands (Vrticka et al., 2012a).

A more consistent and opposite trend is associated with *anxious attachment style*, where increased activation of the aversion system has generally been found in response to negative social cues. In our own study where faces with different expressions were presented as a social feedback signal about performance during a perceptual game (Vrtička et al., 2008), we showed that the amygdala was selectively activated when an angry face was presented as negative feedback after incorrect response (representing social punishment), and the magnitude of this response was correlated with the degree of anxious attachment in participants (Figure 2C). Such increase in amygdala activation is likely to reflect the tendency of anxiously attached individuals to experience heightened distress in situations of personal failure or social disapproval, when social support would be desired instead. In keeping with this notion, another study reported an increase of amygdala activity in response to negative sentences with attachment-related meaning, which was related to individual attachment insecurity (Lemche et al., 2006). However, the latter study did not examine the distinct prototypes or dimensions of attachment using standard structured interviews, but rather inferred general attachment differences (secure or insecure) based on reaction times to the sentences (slow or fast). In addition, in the study by Dewall et al. using the cyberball game (Dewall et al., 2011), increased activation was observed in anterior insula and dorsal ACC during social rejection as a function of anxious attachment style scores, mirroring an increased sensitivity to negative social clues related to social exclusion. Finally, another fMRI investigation also found an enhanced hippocampus response when listening to own *versus* unknown baby cries in mothers who scored low on a maternal care measure (Kim et al., 2010b), and the same brain area exhibited a reduction in gray matter volume which (negatively) correlated with anxious attachment scores (Quirin et al., 2010). These data accord with the role of the hippocampus in stress responses (Foley and Kirschbaum, 2010).

These findings also converge with other, more clinical fMRI investigations assessing attachment orientations and brain activation patterns by means of the adult attachment projective (AAP)—schematic drawings of attachment-related scenes depicting either one or two persons—which distinguishes between a resolved *versus* unresolved attachment orientation (Buchheim et al., 2006, 2008). The latter studies revealed a positive relation between unresolved attachment and activation in both the amygdala and hippocampus to traumatic AAP images in general, as well as an increase in ACC activity in BPD patients—who are considered to have an unresolved attachment style.

In sum, the available data from recent neuroimaging research points to a higher sensitivity to negative social clues, and enhanced recruitment of social aversion or threat systems in relation to anxious attachment, in agreement with previous work suggesting higher vigilance to social emotional cues and hyper-activating of secondary attachment-related strategies in these individuals (Mikulincer and Shaver, 2007). This also converges with behavioral findings showing that anxious attachment correlates with higher arousal and lesser control reported for scenes with negative (sad or threatening) social content (Rognoni et al., 2008; Vrticka et al., 2012a), and that stressful task conditions produce abnormal cortisol responses in anxiously attached individuals, suggesting impaired regulation of the HPA stress system (Kidd et al., 2011). By contrast, avoidantly attached individuals show normal cortisol responses and are thought to use de-activating strategies when processing social emotional information. Note that, although such individual differences may arise in some conditions with no direct attachment-related meaning, there is evidence for relatively specific or stronger effects of AAS on responses to the social significance of events at both the neural (Vrtička et al., 2008; Vrticka et al., 2012a) and behavioral levels (Vrticka et al., 2012b), indicating that differential responses observed in social approach or aversion networks are not merely related to a general modulation of these systems to any emotional challenge. Taken together, such data add support to the view that individual differences in attachment and social affective behaviors may ultimately result from an interaction between genetic factors and learning through early life experiences, but also have extended influences on other emotional contexts beyond interpersonal relationships, as postulated by recent developments in AT (Mikulincer and Shaver, 2007).

## Cognitive versus emotional mentalization

Besides the notion of a basic level of automatic appraisals of safety *versus* danger, underlying social approach *versus* aversion tendencies (Porges, 2003), another important component of social cognition involves a set of more controlled processes mediating conscious representations about others, as well as behavioral regulation and decision making (Lieberman, 2007). In line with this, Fonagy and Luyten (Fonagy and Luyten, 2009) suggested to make a basic distinction between two aspects of social processing necessary to understand and respond to others, which they conceptualized as emotional *versus* cognitive mentalization processes. According to this distinction, *emotional mentalization* would correspond to the rather automatic, implicit, or even unconscious processing of externally-focused (physical and visible) information about others (such as expressions, actions, etc.), which are also closely related to neurocognitive mechanisms implicated in “emotional contagion” (Shamay-Tsoory et al., 2009) or “empathizing” (Baron-Cohen, 2009). Thus, this level of processing implies a predominantly affective representation of other people and events in the world that would correspond to differential activation patterns in the social approach and aversion systems as described above. Consequently, this component of *emotional mentalization* globally overlaps with *affective evaluation processes* in our model (see Figure 1).

In contrast, distinct brain networks are known to be activated by more explicit and voluntary levels of social and affective processing (Lieberman, 2007; Fonagy and Luyten, 2009). These processes are also preferentially involved in the representation of internally-focused information about others (such as mental states, intentions, etc.), and correspond to what Fonagy and Luyten (Fonagy and Luyten, 2009) referred to as a *cognitive mentalization* system. The latter is thought to comprise (mainly but not exclusively) a wide network of areas in the lateral prefrontal (PFC), OFC, and posterior cingulate cortex (PCC), as well as the precuneus, superior temporal sulcus (STS), and temporo-parietal junction (TPJ), plus specialized sensory regions in superior temporal gyrus, lateral occipital cortex, and fusiform cortex (Lieberman, 2007; Fonagy and Luyten, 2009).

Importantly, there is evidence that activity in these two systems for emotional evaluation and cognitive mentalization might also be in a dynamic balance, and that this equilibrium might be strongly influenced by stress factors (Mayes, 2000, 2006). Thus, the higher the stress (arousal), urgency, or novelty of a situation, the more the “switch point” between different modes of processing might be shifted toward an activation of the emotional evaluation system (Fonagy and Luyten, 2009). This shift would correspond to behavioral changes “*from flexibility to automaticity, … that is from relatively slow executive functions … to faster and habitual behavior … ”* (Fonagy and Luyten, 2009; p. 1367). From an evolutionary perspective, such a shift between processing modes would normally be adaptive in threatening conditions, as it can promote immediate and automatic (reflexive) self-protective reactions. However, in interpersonal settings where cognitive mentalization is a necessary prerequisite and danger neither vital nor immediate (Dunbar, 1998), a too strong or exclusive reliance on affective evaluation might represent an insufficient or inappropriate strategy. Crucially, individual differences in AAS might play a key role in adjusting this balance between cognitive and emotional mentalization, in addition to modulating the differential recruitment of approach or aversion tendencies within the affective system itself. According to this view, an anxious attachment style would facilitate emotional mentalization due to a decreased recruitment of cognitive mentalization capacities, whereas an avoidant attachment style would be associated with a predominant use of cognitive mentalization and a suppression of emotional evaluation, at least until the point where such de-activating strategies fail and highly emotionally reactions occur in avoidantly attached individuals (Fonagy and Luyten, 2009).

In this “mentalization-based approach” described by Fonagy and Luyten (2009), no distinction is made between cognitive mentalization in terms of theory of mind (the representation of the internal states of others or oneself) *versus* cognitive control of emotions and social behaviors (regulation), in relation to others or oneself. However, AT suggests that individual attachment styles also imply distinct modes of emotion regulation strategies (Mikulincer and Shaver, 2007), which have no direct relation to mentalization processes. Moreover, an influential theory of emotion regulation theory (Gross, 1998, 2002) has emphasized different types of strategies (e.g., antecedent- and response-focused), but the latter do not make specific reference to interpersonal emotion situations. It therefore appears useful to consider neural networks for theory of mind/mental state representation and emotion regulation/cognitive control separately, in order to understand the effects of attachment style on mentalization and social behaviors.

### Mental state representation

The notion that attachment-related thoughts can modulate brain systems involved in the representation of others' mental states has received some support from pioneer work examining the neural substrates of romantic love, measuring brain responses to faces of partners *versus* friends *versus* unknown persons (Zeki, 2007). These studies reported consistent deactivations in cortical brain areas known to be involved in theory of mind (Zeki, 2007), accompanied with increased activity in the affective evaluation (emotional mentalization) networks (Gobbini and Haxby, 2007; Lieberman, 2007), supporting the view of a reciprocal balance between cognitive and emotional mentalization processes. Furthermore, mothers viewing infant stimuli have also been found to exhibit greater activity in superior medial PFC regions (BA 8, 9, and 10) involved in cognitive mentalization (Swain et al., 2007), which was interpreted as reflecting the capacity of these mothers “*to orchestrate a new and increased repertoire of complex interactive behaviors with infants … ”* (Kim et al., 2010a; p. 698). Thus, both increases and decreases may arise in different parts of the cognitive mentalization networks. However, still little is known about whether, and how, these networks might be influenced by specific adult attachment orientations (e.g., secure or insecure) in different individuals.

One study relevant to this issue examined gray matter volume and brain activations to own infant cries in mothers in the early postpartum period, who were divided into two groups according to their perceived maternal care scores (Kim et al., 2010b), a measure reflecting differences along secure *versus* insecure attachment dimensions. Not only did the mothers with high perceived maternal care scores display increased gray matter volume in several areas associated with theory-of-mind, such as the PFC (superior frontal and orbital gyrus; BA 10 and 47), STS, and fusiform gyrus, but they also showed increased BOLD signal change in these areas when hearing baby cries. These results suggest that mothers with secure attachment traits (high scores on perceived maternal care) might more readily engage in complex social behaviors involving mentalization and theory of mind when interacting with children, possibly implying more efficient cognitive processing to represent their mental states in terms of intentions or needs. In turn, this could potentially have beneficial effects on the emerging attachment styles of the child him/herself (see below). Conversely, low scores on the perceived maternal scale reflecting insecure attachment, were associated with increased hippocampus responses to infant cries in the same mothers. As the hippocampus is known to play an important role in stress responses (Foley and Kirschbaum, 2010), this pattern again nicely reflects the notion of a balance between cognitive mentalization and emotional evaluation processes. Moreover, it also provides support to the view that a secure attachment style may facilitate the access to mental state representations, whereas an insecure attachment may lead to more emotional mentalizing. However, no finer distinction between avoidant and anxious attachment styles was made in this study.

Other researchers (Buchheim et al., 2008) explored brain responses during exposure to monadic *versus* dyadic pictures of the AAP. This study compared healthy individuals with resolved and unresolved profiles, as well as patients with BPD who typically exhibit an unresolved attachment orientation, for example as a result of traumatic attachment-related experiences in childhood. The results revealed that only BPD patients—but not unresolved controls—displayed increased activity in the STS when exposed to dyadic images of the AAP. Simultaneously, BDP patients showed strongest reports of affective loss and abuse, relative to the unresolved controls. Because the STS is a key substrate of the theory-of-mind network, and BPD patients are known to show distorted and “hyperanalytical” thinking in attachment contexts, possibly reflecting enhanced representation to other's mental states, this pattern of findings was interpreted as “*a neural indicator of fear-based hypervigilance in attachment relationships …* ” in BPD patients (Buchheim et al., 2008; p. 233). These data therefore indicate that, in some cases, mental state representations may also be enhanced by attachment insecurity, and that this might be more strongly associated with hypervigilance linked to attachment anxiety (fear caused by trauma). Moreover, because in unresolved BPD patients, increased emotional mentalizing was also demonstrated by the same study (see above), the notion of an obligatory “push-pull” between emotional and cognitive mentalization as proposed by Fonagy and colleagues (Fonagy and Luyten, 2009) may not always hold true. It remains to be seen whether distinct aspects of mental state representations are differentially modulated by anxious and avoidant attachment traits. In particular, some theorists (Mikulincer and Shaver, 2007) have proposed that anxious and avoidant attachment dimensions might correspond to different access to positive and negative representations of others (as well as of the self). Preliminary data from our own ongoing work provide tentative support to these models.

In sum, the emerging evidence from neuroscience research regarding the impact of individual differences in AAS on mental state representation seems to suggest that an insecure attachment orientation may not always lead to decreased use theory of mind and controlled appraisals of mental states in others, but could also have inverse effects, particularly in the case of attachment anxiety (hypervigilance). This somewhat contradicts the hypothesis put forward by the developmental mentalization-based approach of Fonagy and Luyten (Fonagy and Luyten, 2009), and their proposal that attachment insecurity observed in BPD patients reflect a lower cognitive mentalization combined with higher emotional mentalization. However, as BPD is mainly associated with an unresolved attachment orientation, whereas both anxious and avoidant attachment actually fall into a resolved category, a strict opposition between cognitive and emotional mentalization should be regarded with caution when using it from a psychological perspective in healthy people (adults), rather than in a psychopathological context like BPD. This apparent discrepancy might also be explained by the fact that Fonagy and Luyten (2009) considered a single social cognitive system for controlled mentalization, whereas a more complete push-pull model should regard theory of mind and emotion regulation as separate components of cognitive mentalization processes, and the latter might be more strongly influenced by attachment insecurities in different directions (see next section below). More research using a neuroscientific approach is still needed to clarify these issues, particularly regarding attachment avoidance, which is conceptualized as involving increased cognitive mentalizing up to a certain “breakdown threshold”.

### Emotion regulation

Attachment theory assumes that a key component of individual differences in attachment styles involves distinct affective regulation strategies leading to hyper- or hypo-activation of attachment system in anxious and avoidant people, respectively. The relation of these regulation strategies to other mechanisms of emotion regulation is still incompletely elucidated, however. Both the cognitive mechanisms and the neural substrates of emotion regulation have been extensively investigated in the past decade (Gross, 1998, 2002; Ochsner and Gross, 2005), but in conditions totally unrelated to attachment. In fact, most of this work has focused on emotion experience at the *intra*personal level rather than in *inter*personal or social contexts. Traditionally, a main distinction has been made between so-called antecedent- and response-focused emotion regulation strategies. The former aims at interfering early with the emotion generation process, before the emotional response arises, through distraction from an emotional event or modulation of its meaning by extrinsic information. The second type of regulation instead is characterized by a reaction to an already elicited emotion, implying voluntary control of subsequent behavior. Another important difference between these two forms of regulation concerns the underlying mechanisms. Antecedent-focused emotion regulation is thought to operate either through attentional processes (i.e., avoiding exposure to the emotion-eliciting stimulus using distraction techniques, etc.), or cognitive re-appraisal strategies (i.e., changing the interpretation of the stimulus and/or minimizing its emotional significance). Hence, these emotion regulation strategies are generally referred to as *distraction, detachment*, or *reappraisal*. By contrast, response-focused emotion regulation involves the (voluntary) inhibition or transformation of the emotional response or behavior after an emotion has ben generated (e.g., facial expression or physiological changes). This strategy is therefore generally referred to as *(expressive) suppression*. In terms of brain activity patterns, the employment of both emotion regulation strategies has been linked with increased PFC cortical activation related to executive control and/or behavioral inhibition (parts of the cognitive mentalization network), and a simultaneous modulation (e.g., reduction) of responses evoked in the emotional evaluation system (Ochsner et al., 2002, 2004b; Ochsner and Gross, 2005; Kim and Hamann, 2007; McRae et al., 2010; Vrticka et al., 2011). In addition, there is a general consensus that, on the long term, antecedent-focused emotion regulation may represent a more beneficial emotion regulation strategy (Gross, 1998, 2002).

Attachment theory makes several claims about the efficiency as well as the preferential use of different emotion regulation strategies. On the one hand, secure attachment is associated with a constructive and thus successful use of antecedent-focused emotion regulation, mainly by means of cognitive re-appraisal, leading to a low and stable emotional responsiveness in stressful social situations. On the other hand, insecure attachment is generally associated with difficulties in emotion regulation capacities, leading to poor outcomes in stressful social situations and persisting high emotionality. In particular, attachment avoidance may lead to preferential use of response-focused emotion regulation through suppression, which can reduce overt emotional reactions, but is not very efficient in regulating emotion elicitation itself. Moreover, suppression may work up to a certain point only, after which this protective mechanism will break down and avoidantly attached people become overwhelmed by their emotions. In contrast, attachment anxiety has not been associated with consistent patterns according to the classic emotion regulation framework (Mikulincer and Shaver, 2007). Although social emotional hypervigilance typically observed in anxiously attached individual might be driven by a persistent cognitive up-regulation of affective processing through re-appraisal, and/or some deficiency in inverse mechanisms aiming at decreasing emotional reactivity, the exact functioning of these processes and their modulation by attachment anxiety remains to be elucidated (Mikulincer and Shaver, 2007; Fonagy and Luyten, 2009).

Recent neuroimaging investigations have provided new support to the notion that *attachment insecurities* are generally associated with less efficient or even disturbed emotion regulation capacities. In a first study of this kind, Coan et al. (Coan et al., 2006) scanned female participants while holding their husband's hand and being threatened with electrical shocks. They found that the higher the marital quality reported by the participants (reflecting a secure attachment in current romantic relationship), the less activity in PFC cortical areas as well as anterior insula and hypothalamus there was during shock anticipation, suggesting more efficient emotion regulation capacities. Conversely, another study using items of the AAP as visual stimuli (Buchheim et al., 2006) found that only participants with unresolved attachment displayed increased activation in lateral PFC areas plus amygdala and hippocampus as a function of increasing traumatic image content, reflecting impaired emotion regulation capacities. Finally, a third study used an emotion-word Stroop task during which participants had to indicate the color of unpleasant, neutral, or pleasant words while ignoring their meaning (Warren et al., 2010). The results revealed that an insecure attachment style was associated with poor task performance and simultaneously high activity in both dorsolateral PFC and OFC, again pointing to less efficient cognitive control capacities—here more specifically linked with a vulnerability to distraction by attachment-relevant emotional information.

Other investigations focused on the distinction between different insecure attachment orientations. An early study by Gillath and colleagues (Gillath et al., 2005) used fMRI in participants who were told to either think or stop thinking about negative relationship scenarios. Their findings revealed that *anxiously attached participants* exhibited increased activity in the ATP, hippocampus, and dorsal ACC when thinking about negative emotions, but less activity in the OFC when suppressing such thoughts. Moreover, activity in ATP and OFC was inversely correlated. This suggests a stronger recruitment of neural systems involved in negative emotional states during normal processing of attachment-related information, and impaired regulatory capacities to inhibit such processing, consistent with the hallmarks of anxious attachment. Conversely, high scores on *avoidant attachment* were associated with sustained activity in subcallosal cingulate and medial frontal gyrus (BA 9) during both the “think” and the “don't think” conditions, which was interpreted as a failure of “task-induced deactivation” (Gillath et al., 2005)—but could actually also be understood as persistent unsuccessful inhibition. However, these results provide only indirect evidence for altered emotion regulation capacities in attachment anxiety and avoidance.

**Figure 3:**
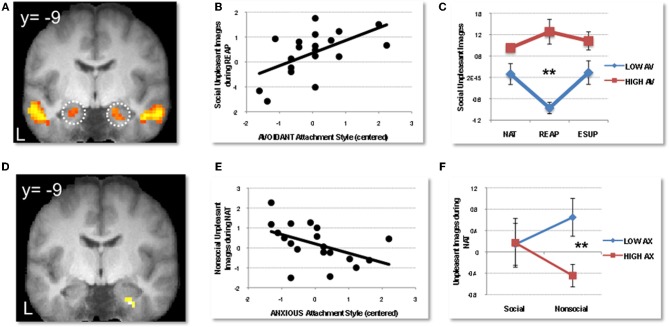
**Modulation of social emotion perception and regulation by adult attachment style.** Adapted from Vrticka et al. (2012a). **(A)** Bilateral amygdala activation to social (versus nonsocial) emotional scenes perception, regardless of valence (positive or negative) and task. **(B)** Positive correlation between avoidant attachment (AV) scores and activity in left amygdala for social negative images during reappraisal (averaged across voxels). **(C)** Median split illustrating data for high (red; *N* = 5) *versus* low (blue; *N* = 8) avoidantly attached participants in left amygdala, showing a decrease in activity to social negative images during reappraisal for the low but not high avoidant group (^**^indicate the differential response accounting for significant effects in the correlation analysis). **(D)** Right amygdala activation to negative scenes showing a significant modulation by anxious attachment (AX) scores during natural viewing conditions. **(E)** Negative correlation between anxious attachment scores and response to nonsocial negative scenes in the right amygdala. **(F)** Median split illustrating data for high (red; *N* = 8) versus low (blue; *N* = 9) anxiously attached participants in right amygdala, showing that activation to negative scenes was greater for nonsocial content in those with lower AX scores, but greater for social content in those with higher AX scores. (^**^indicate the differential response accounting for significant effects in the whole-brain correlation analysis). NAT = natural viewing, REAP = reappraisal, ESUP = expressive suppression. BOLD signal is depicted in arbitrary units, and error bars represent +/− 1 standard error from mean.

We recently extended these findings by specifically comparing the effect of both reappraisal and suppression strategies within the same fMRI experiment (Vrticka et al., 2012a). For this purpose, participants were shown social or nonsocial visual scenes, with either a positive or negative content, while being asked to either attend to the scenes naturally (NAT), reappraise their content to diminish any emotional interpretation (REAP), or suppress any visible expression of emotion elicited by the images (ESUP). Distinct patterns of activations were observed as a function of the degree of attachment avoidance or anxiety, but interestingly, the most important differences were disclosed during the spontaneous emotion processing condition (NAT). When participants were instructed not to use any regulation strategy, higher scores on *avoidant attachment* were associated with increased activity for negative social scenes in dorsal and ventral anterior cingulate, as well as in both the lateral and medial dorsal PFC, but for positive social scenes in the medial OFC and supplemental motor area (SMA). This pattern may reflect heightened cognitive and emotional conflict in combination with increased regulatory inhibition during spontaneous viewing of social emotional scenes. Furthermore, during REAP, amygdala activation to negative social images decreased for low, but not high avoidantly attached participants, implying that this emotion regulation strategy was not efficient in reducing affective mentalizing (Figures 3A,B,C). Finally, during SUP, attachment avoidance was associated with stronger responses to positive social images in the SMA and caudate, again implying stronger regulatory efforts with the successful use of suppression. As a whole, these brain imaging data support but also extend the notion put forward by AT (Mikulincer and Shaver, 2007) that attachment avoidance is associated with a preferential use of emotion suppression in interpersonal/social contexts. Furthermore, they reveal that reappraisal may not work for these individuals, leading to impaired down-regulation of amygdala reactivity. This pattern may help understand why avoidantly attached individuals tend to become highly emotional when their preferred regulation strategies fail or cannot be employed.

Conversely, in the same study, higher scores on *attachment anxiety* were correlated with higher amygdala activation to social negative scenes during spontaneous viewing without specific regulation instructions (NAT; Figures 3D,E,F), confirming the previous proposals of heightened emotional mentalizing. However, there was no additional evidence of impaired emotion regulation, neither during REAP, nor during SUP. Moreover, we also found that anxious attachment predicted greater activation in the parahippocampal cortex (during NAT and REAP), suggesting that it might ease the access to memory about previous attachment experiences, as already proposed in a previous study (Gillath et al., 2005). Although no final conclusions can be drawn from such results, our data nonetheless imply that anxiously attached individuals can successfully apply both antecedent- and response-focused emotion regulation strategies when properly instructed to do so, and that the increased emotional mentalization in spontaneous conditions could partly be accompanied with an eased access to memory information, for example about former personal attachment experiences.

Overall, these neuroscientific data on emotion regulation converge with theoretical assumptions that have generally characterized attachment insecurities by altered or less efficient cognitive control capacities (Mikulincer and Shaver, 2007), leading in turn to enhanced emotional responses. Furthermore, these new findings also indicate that the underlying mechanisms may differ in avoidantly and anxiously attached individuals, particularly with respect to the use and/or efficacy of antecedent- or response-focused emotion regulation strategies. However, although extant data have begun to characterize these individual differences in relation to a well-established model of emotion regulation (e.g., making a key distinction between reappraisal and suppression), it remains to be determined whether more specific regulation strategies are differentially modulated by individual attachment orientations—beyond the traditional strategies studied in healthy people (Ochsner et al., 2002, 2004a; Kim and Hamann, 2007; McRae et al., 2010; Kanske et al., 2011; Vrticka et al., 2012a). These issues should be investigated in more detail in the future, because they could prove of great importance to develop and monitor intervention strategies of attachment insecurities and their associated regulation deficits.

## Molecular and genetic mechanisms

As mentioned in the introduction, some investigations on the neurobiological underpinning of (human) social behavior have begun to explore the molecular and genetic mechanisms at play in social affective processing, learning, and bonding (Insel and Young, 2001; Meyer-Lindenberg, 2008; Heinrichs et al., 2009; Champagne, 2010; Insel, 2010; Bakermans-Kranenburg and van Ijzendoorn, 2011; MacDonald and MacDonald, 2011), as well as those implicated in disorders such as autism, sociopathy, or aggression (Piggot et al., 2009; Koenigs et al., 2011; Soyka, 2011).

Within this new field of research, several studies have recently focused on specific questions related to individual differences in attachment style (Gillath et al., 2008; Salo et al., 2008; Costa et al., 2009; Bradley et al., 2011; Troisi et al., 2011). For example, there is emerging evidence that some aspects of attachment are transmitted across generations (Hautamaki et al., 2010; Shah et al., 2010), and that genetic polymorphisms related to emotions and social behavior might influence individual responses to attachment-related experiences during development (Gillath et al., 2008). In particular, anxious attachment has been found to correlate with a polymorphism of the DRD2 dopamine receptor gene, whereas avoidant attachment is associated with a polymorphism of the 5HT2A serotonin receptor gene. By contrast, no relation was found between attachment insecurities and a polymorphism of the oxytocin receptor (OXTR) gene (Gillath et al., 2008), even though the latter has been associated with other individual differences in social behavior (Meyer-Lindenberg, 2008). Future studies will also have to examine possible epigenetic mechanisms, looking for particular gene-environment interactions which can be induced by early life experiences (Graeff et al., 2011). Possible candidate genes comprise, among others, oxytocin, vasopressin, dopamine, the opioids, cortisol, and serotonin (see Figure 1).

Two recent fMRI studies have also described an association between amygdala activations during the perception of emotional facial expressions and OXTR and vasopressin (Avpr1a) receptor-gene polymorphisms, which even correlated with individual measures of emotional reactivity and prosocial temperament in one study (Meyer-Lindenberg et al., 2009; Tost et al., 2010). Future experiments should employ more specific social emotional tasks and probe for modulations by more specific personality traits, including in particular individual differences in attachment style. The effects of genetic or epigenetic factors on distinct neural circuits mediating attachment processes and their impact on social cognition (as depicted in Figure 1) should also be systematically examined.

## Summary and outlook

The present paper proposes an overview of the human brain systems underlying individual differences in AAS, and how they influence social and emotional processing in healthy individuals. It employs a developmental psychological AT perspective and integrates these notions in a schematic functional architecture derived from current neuroscience models, with two major components for affective and cognitive representations, respectively. This framework suggests that early interactions between children and their main attachment figure as well as subsequent social experiences, perhaps combined with some genetic factors, will become integral parts of one's personal schemas guiding relationships with others in later life, resulting in profound individual differences characterized by secure or insecure (avoidant, anxious, disorganized/unresolved) attachment. Such personality traits will produce important and long-lasting influences on social emotional information processing and regulation, associated with differential recruitment of specific functional brain networks for understanding and responding to others in close (or at times less close) relationships.

We propose a functional neuroanatomical model to describe such interactions, which builds on two main core components. These comprise, on the one hand, a system for rapid, automatic affective appraisals (emotional mentalization), which is primarily involved in encoding basic dimensions of safety *versus* threat, or approach *versus* aversion tendencies in social contexts; and on the other hand, a system for controlled social processing and regulation (cognitive mentalization), operating in a more conscious, voluntary mode, which is involved in representing the mental states of others (theory of mind) and regulating one's own behavior, thoughts, and emotions. These two functional components rely on distinct brain networks (Porges, 2003; Lieberman, 2007; Fonagy and Luyten, 2009), essentially centered on limbic cortico-subcortical areas (e.g., amygdala, striatum, insula, cingulate, hippocampus) for affective evaluations, and fronto-temporal areas (e.g., MPFC, OFC, STS, TPJ, etc.) for cognitive mentalization and regulation, respectively. Importantly, these components may entertain a reciprocal dynamic balance between each other. Moreover, their differential recruitment across individuals in social contexts allow for a distinction between behaviors and emotions associated with specific attachment orientations (avoidance or anxiety), rather than just a distinction between secure *versus* insecure or disorganized/unresolved attachment.

According to this model, an *avoidant attachment style* is characterized by blunted responses in both subparts of the emotional mentalizing systems (signaling social safety or threat), reflecting a deactivation of attachment needs. Findings regarding mental state representation specifically related to attachment avoidance are still preliminary and need further confirmation. Yet, avoidant attachment style also appears to imply a distinctive pattern of emotion regulation strategies, with greater reliance on suppression but difficulties in using reappraisal (antecedent-focused regulation) to dampen affective appraisals.

In contrast, an *anxious attachment style* is associated with enhanced responses to social emotional information signaling threat and motivating aversion, mirroring the hyperactivation of attachment needs and subjective lack of control observed behaviorally. This might also be combined with enhanced recruitment of mental state attribution systems in some situations related to fear (trauma in BPD patients). However, emotion regulation appears relatively operational when explicitly required by task instructions, although mingled with greater recruitment of associative memory systems that may promote access to memories of previous attachment contexts.

This research highlights the fact that social interactions and emotions therein are susceptible to strong modulations by individual differences, reflecting (among others) the key role of the attachment history of a person as well as possible neurobiological predisposition factors. Such consideration of individual attachment style and history in recent neuroimaging studies appears critical to extend social and affective neuroscience research to a comprehensive and valid framework of socially motivated behaviors, although there still is a lack of experimental investigations of these effects in more complex and “true” social interactions. Future studies should therefore aim at better assessing attachment effects on brain responses during “real” social encounters, or at least in laboratory context resembling the latter as closely as possible, as for example by employing functional near infrared spectroscopy (fNIRS) in two participants at the same time, also referred as to hyperscanning (Cui et al., 2012).

In addition, despite the fact that most attachment effects described in this review concern healthy non-clinical populations, they also have implications for promoting well-being and reducing social stress, and may in addition provide useful clues regarding attachment system dysregulations in patients with psychopathologies or abnormal social behaviors (Galynker et al., 2011; Nolte et al., 2011). Future investigations need to deepen our knowledge of the neural mechanisms involved in different facets of attachment, its development (brain activation patterns related to attachment in childhood and adolescence and their transition into adulthood) and its malleability by new experiences and learning, including at the level of gene-environment interactions. We believe that this endeavor will be made possible by using an interdisciplinary approach based on neuroimaging, genetic, and psychological investigations in humans, as well as innovative studies on animal models of social behaviors, as effectively illustrated by many recent advances in social neuroscience.

### Conflict of interest statement

The authors declare that the research was conducted in the absence of any commercial or financial relationships that could be construed as a potential conflict of interest.
